# Metabolic syndrome, high-sensitivity C-reactive protein and the risk of heart failure: the Kailuan cohort study

**DOI:** 10.3389/fendo.2025.1544823

**Published:** 2025-04-22

**Authors:** Yan Tian, Yanxiu Wang, Dandan Zhao, Huayu Sun, Hao Wu, Peng Yang, Shouling Wu, Ying Wu, Shuohua Chen, Yun Li

**Affiliations:** ^1^ School of Public Health, North China University of Science and Technology, Tangshan, China; ^2^ Department of Cardiology, Kailuan General Hospital, Tangshan, China; ^3^ Department of Neurosurgery, Affiliated Hospital of North China University of Science and Technology, Tangshan, China; ^4^ Tangshan Key Laboratory of Clinical Epidemiology, Tangshan, China; ^5^ Hebei Key Laboratory of Occupational Health and Safety for Coal Industry, Tangshan, China

**Keywords:** metabolic syndrome, C-reactive protein, heart failure, risk factors, cohort study.

## Abstract

**Background:**

Metabolic syndrome (MetS) and elevated high-sensitivity C-reactive protein (hs-CRP) have been identified as risk factors for heart failure (HF) in some studies. However, little was known about the co-exposure of MetS and inflammation to HF. We aimed to investigate the combined effect of MetS and high hs-CRP levels on the risk of incident HF.

**Methods:**

The study included 94,841 participants without HF selected from the Kailuan cohort in 2006 (the baseline) and then followed up until 31 December 2020. Participants were divided into four groups based on the presence of MetS and high hs-CRP levels (>3mg/L) at baseline: MetS-CRP- (n=53,937), MetS-CRP+ (n=10,338), MetS+CRP- (n=23,521), MetS+CRP+ (n=7,045). Cox regression models were used to analyze the association of MetS and inflammation with the risk of HF. Statistical significance was defined as a two-tailed P value < 0.05.

**Results:**

The mean age of the participants was 51.5 ± 12.5 years, and 75,976 (80.0%) were male. During 13.1 years of follow-up, 3,058 participants were diagnosed with HF. The HF incidence rate of four groups were 1.69/1000pys, 2.95/1000pys, 3.27/1000pys, 5.33/1000pys. The HR for MetS-CRP+, MetS+CRP-, and MetS+CRP+ were 1.29 (95% CI, 1.15-1.45), 1.40 (95% CI, 1.29-1.53), and 1.85 (95% CI, 1.65-2.06), respectively, compared with MetS-CRP-. After stratification by age (p for interaction < 0.01), compared with the MetS-CRP- group, the HR of the MetS+CRP+ group was 2.17 (95% CI, 1.83-2.57) in participants with < 60 years and 1.53 (95% CI, 1.32-1.78) in participants with ≥ 60 years. There was an interaction between groups and ues of antihypertension medication (p for interaction <0.01). Compared with MetS-CRP-, the risk of HF in the MetS+CRP+ group was increased 1.38-fold (95% CI, 1.12-1.70) in participants with antihypertension medication use and 2.00-fold (95% CI, 1.75-2.27) in participants without antihypertension medication use.

**Conclusions:**

The combination of MetS and elevated hs-CRP was associated with increased risk of HF in the Chinese population.

**Clinical trial registration:**

https://www.chictr.org.cn, identifier ChiCTR-TNRC-11001489.

## Introduction

Heart failure (HF) is the end-stage of cardiac dysfunction. According to the Global Burden of Disease (GBD), the global number of HF cases increased from 33.5 million in 1990 to 64.3 million in 2017 (1). In China, the number of people with HF was approximately 12.1 million, and this number was expected to increase in the future (2). A large cohort study reported that all-cause mortality after hospital discharge in Chinese patients with HF was 28.2% at 3 years (3). Many studies suggested an association between the increasing prevalence of HF and factors such as hypertension, diabetes mellitus, cigarette smoking and other unhealthy lifestyle factors (4, 5). However, the causes of HF were not fully understood. The development of the disease is usually influenced by the co-occurrence of multiple adverse factors, and little research has been conducted on the co-occurrence of these factors. Therefore, a joint evaluation of risk factors for HF explores the pathogenesis of HF, and provides crucial evidence for the prevention of HF onset.

Metabolic syndrome (MetS) is a cluster of metabolic disorders including obesity, hypertension, hyperlipidemia and hyperglycemia. It has a high prevalence in both developed and developing countries (6). The pathogenesis of HF caused by MetS was related to visceral fat accumulation, insulin resistance, and neuroendocrine system activation. For example, activation of the neuroendocrine system caused an increase in blood pressure and heart rate, and the heart used compensatory contractions to meet the blood supply (7). However, prolonged cardiac compensation caused hypertrophy of cardiomyocytes, leading to ventricular remodelling and progression to HF. A large cohort study in Korea found an association between MetS and an increased risk of HF (8). High-sensitivity C-reactive protein (hs-CRP) is a widely used clinical marker to assess inflammation, particularly in the assessment of cardiovascular disease risk and prognosis. Inflammation plays a key role in the development of HF. According to Pearson TA et al, hyperactivation of inflammatory factors led to an imbalance in calcium metabolism in cardiomyocytes, resulting in cardiomyocyte apoptosis and necrosis (9). Recent research found that the interaction between metabolic disorders and inflammation created a vicious circle. For example, the release of inflammatory cytokines interfered with peripheral insulin signaling pathways, causing the body to become less sensitive and responsive to insulin, which led to insulin resistance and impaired glucose metabolism (10). Adipocyte expansion caused by metabolic disorders led to reduced levels of anti-inflammatory factors, resulting in systemic inflammation (11). Several studies showed that the combination of MetS and inflammation increases the risk of atrial fibrillation (AF) and gastrointestinal tumours (10, 12–14). However, little was known about the co-exposure of MetS and inflammation to HF. Therefore, we used data from the Kailuan study cohort to systematically analyze the association of MetS and elevated hs-CRP levels with the incident HF.

## Methods

### Study design and population

**Figure 1:**
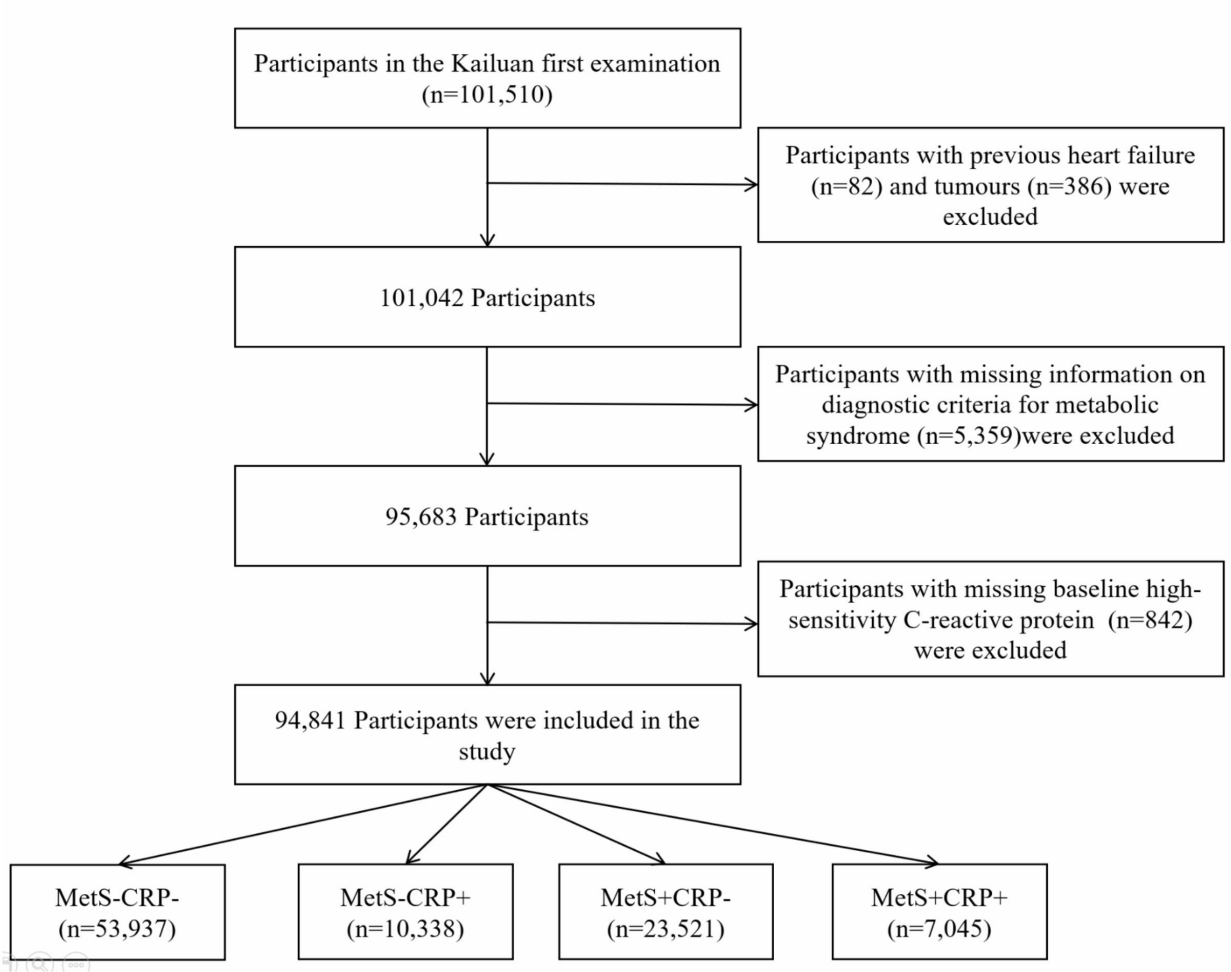
Flow chart of the study.

The Kailuan cohort (registration number: ChiCTR-TNRC-11001489) was a large prospective cohort study conducted in the Kailuan group, in Tangshan City, Hebei Province, China. Detailed information on the study design and methods has been previously documented in published reports (15, 16). The initial examination of 101,150 adult participants from the active and retired population of Kailuan Group took place between 2006 and October 2007. They were then followed up every two years with standardized questionnaires, clinical examinations and laboratory tests. **Figure 1** showed the study procedure. Participants with a history of HF (n=82), a history of cancer (n=386), missing information on diagnostic criteria for MetS (n=5,359), missing baseline information on hs-CRP (n=842) were excluded. In the end, 94,841 participants were included in the study.

All participants provided written informed consent. The study was performed according to the guidelines of the Helsinki Declaration and was approved by the Ethics Committee of Kailuan Hospital (Ethics approval number:200605).

### Data collection

The baseline data were obtained at the first physical examination between 2006 and 2007. Height was measured using a tape measure to an accuracy of 0.1 cm, and weight was measured using a calibrated weight scale to an accuracy of 0.1 kg. Waist circumference (WC) was measured with a tape measure, using the midpoint between the lower edge of the ribs and the upper edge of the hips as the reference point. Blood pressure (BP) was measured twice consecutively with a mercury sphygmomanometer with the participant in an upright sitting position after a 5-minute rest. The mean of these two BP readings was recorded for subsequent analysis. If the difference between the two readings exceeded 5 mm Hg, a third reading was taken and the average of all three readings was used for data analysis. A standardized questionnaire was used to collect information on various baseline characteristics, including sex and date of birth, and lifestyle factors such as smoking status, alcohol consumption, salt intake and physical activity. In addition, personal or family medical history was documented, including conditions such as hypertension, diabetes mellitus and cardiovascular disease. Information on medication use, including antihypertensive, hypoglycemic and lipid-lowering medications, was also recorded.

### Laboratory examinations

Blood samples were collected from the antecubital vein in the morning after an overnight fast. Fasting blood glucose (FBG) was measured by the hexokinase/glucose-6-phosphate dehydrogenase method. Triglycerides (TG) were measured by an enzymatic method, and high-density lipoprotein cholesterol (HDL-C) concentration was measured by a direct method. Uric acid (UA) was measured using a commercial kit from Kewa Bioengineering, Shanghai, China. Hs-CRP levels were measured using a highly sensitive immunoturbidimetric assay (Cias Latex CRP-H, Kanto Chemical Co. Inc, Tokyo, Japan) with a detection limit of 0.1 mg/L. Biochemical indices were measured in the central laboratory of Kailuan General Hospital using a Hitachi 7600 autoanalyzer.

### Metabolic syndrome definition and subgroups

MetS was defined according to the criteria established by the ATP III criteria, as follows: The presence of three or more of the following factors: 1) SBP≥ 130 mm Hg or DBP ≥ 85 mm Hg or use of antihypertensive medication, 2) FBG ≥ 5.6 mmol/L or use of hypoglycemic medication, 3) TG ≥ 1.69 mmol/L, 4) HDL-C < 1.04 mmol/L in men and < 1.29 mmol/L in women, or using lipid-lowering medication, and 5) WC ≥ 85 cm in men, WC ≥ 80 cm in women (12, 17). High hs-CRP levels were defined as serum hs-CRP > 3 mg/L (9).

Participants were divided into four groups according to the presence or absence of MetS and hs-CRP levels: 1)MetS-CRP-, participants without MetS and with hs-CRP levels ≤ 3 mg/L. 2) MetS-CRP+, participants without MetS and with hs-CRP levels > 3 mg/L. 3) MetS+CRP-, participants with MetS and hs-CRP levels ≤ 3 mg/L. 4) MetS+CRP+, participants with MetS and hs-CRP levels > 3 mg/L.

### Definition of study outcomes

The study started with the first physical examination in 2006, and the primary outcome was the first diagnosis of HF. The follow-up end point was 31 December 2020 for individuals not experiencing the event. The end point was defined as the time of death if death occurred during follow-up. Discharge records from 11 local hospitals were collected and reviewed annually by specialized teams to identify patients with suspected HF. The definition of HF was based on the Chinese Guidelines for the Diagnosis and Treatment of HF 2018 (18). Diagnostic parameters included clinical presentation and laboratory tests. The diagnosis of HF was confirmed by the presence of (1) and any of (2) or (3): (1) symptoms of HF, including dyspnea, fatigue, and fluid retention, and a discharge diagnosis of central function classified as NewYork Heart Association cardiac function classes II, III, IV or Killip II, III, IV; (2) left ventricular ejection fraction ≤ 50%, as measured by Simpson’s method modified by 2-dimensional and Doppler echo cardiography; and (3) elevated plasma NT-proBNP (N-terminal pro-B-type natriuretic peptide) levels.

### Assessment of covariates

Smoking was defined as an average of at least 1 cigarette per day in the past year, divided into non-smokers and current smokers, with non-smokers including ex-smokers. Alcohol consumption was defined as drinking ≥ 100 ml of liquor (with an alcohol content of 50% or more) per day on average in the past year, divided into those who still drink alcohol and those who do not, and those who do not include those who have stopped drinking. Active physical activity was defined as exercising at least 3 times a week for at least 30 min each time, and a high-salt diet was defined as a salt intake of 10 g or more per day. Educational level was categorized as junior high school and below, and high school and above. Pre-diabetes was defined as FBG at 5.6-6.9 mmol/L (19). Hypertension was defined as self-reported history of hypertension, current treatment with an antihypertensive agent or a measured SBP ≥ 140 mmHg and/or DBP ≥ 90 mmHg. Diabetes was defined as a self-reported history of diabetes, current treatment with a hypoglycemic agent or a FBG ≥ 7.0 mmol/L. Dyslipidemia was defined as meeting any of the following criteria: total cholesterol (TC) ≥ 6.22 mmol/L, TG ≥ 2.26 mmol/L, low-density lipoprotein cholesterol (LDL-C) ≥ 4.14 mmol/L, HDL-C < 1.04 mmol/L, or self-reported history of hyperlipidemia. The estimated glomerular filtration rate (eGFR) was calculated according to the formula of the Chronic Kidney Disease Epidemiology Cooperation (CKD-EPI) (20).

### Statistical analyses

Normally distributed continuous variables were expressed as mean ± SD and compared using the ANOVA. Skewed distribution continuous variables were expressed as median (P25-P75) and compared using the Kruskal-Wallis test. Categorical variables were expressed as percentages and compared using the chi-squared test. Cox proportional hazards models were used to analyze the association of MetS and components with HF and the association of hs-CRP (grouped by 3 mg/L) with HF and the association of the combination of MetS and hs-CRP with HF. The model was adjusted for age, sex, smoking, alcohol consumption, physical activity, education, salt intake, family history of cardiovascular disease, eGFR, UA, use of antihypertensive medication, use of hypoglycemic medication, and use of lipid-lowering medication. Subgroup analyses were stratified by age (< 60 years and ≥ 60 years) and gender. To examine the effect of antihypertensive medication, stratification was performed according to the use and intensity of antihypertensive medication.The incident rate of HF was calculated using the number of HF occurrence divided by 1000 person-years. The cumulative incidence of HF in the different groups was calculated using the Kaplan-Meier method. The log-rank test was used to compare groups. To explore the effect of MetS and hs-CRP interaction on HF, we included MetS, hs-CRP and their multiplicative interaction terms in the Cox model after adjustment for covariates and calculated the excess relative risk (RERI), attributable proportion (AP) and synergy index (SI) to estimate the additive interaction. Data were analyzed using SAS 9.4 (SAS Institute, Cary, North Carolina) statistical software, and statistical significance was defined as a two-tailed P value < 0.05.

### Sensitivity analysis

To ensure the robustness of the results, we separately excluded participants who had myocardial infarction (MI), AF before the onset of HF. To avoid the possibility of different definitions of MetS affecting the results of the study, we repeated the analyses using the definition of MetS from the IDF criteria (21). To assess the effect of different inflammation degrees, the sensitivity analysis was performed by re-grouping with CRP 2 mg/L as the cut-off point to verify the reliability of the results (22). To avoid the effects of acute inflammation and infectious diseases, we excluded individuals with hs-CRP > 10 mg/L and repeated analyses (9, 23). Considering that patients with hypertension, diabetes and dyslipidemia may contribute to differences in results, we excluded patients with pre-diabetes, hypertension, diabetes and dyslipidemia from the baseline. Taking into account the effect of treatment on outcome, we excluded participants who received pharmacological treatment (antihypertensive, hypoglycemic, or lipid-lowering drugs). Considering that the progression of non-MetS participants to the MetS population at follow-up had an impact on the results, we excluded participants without MetS at baseline who developed MetS at follow-up (24). Considering the effects of age on outcome, we used participants in the MetS+CRP+ group as the exposure group, and matched three control groups by age (± 1 year) among participants in the MetS-CRP- group, MetS-CRP+ group, and MetS+CRP- group, respectively.

## Results

### Participant characteristics

A total of 94,841 participants (mean age 51.5 ± 12.5 years) were recruited, of whom 75,976 (80.0%) were male. The baseline characteristics of the four groups were shown in **Table 1**. Statistical differences between the four groups were found for sex, age, SBP, DBP, UA, WC, Tg, Tc, LDL-C, HDL-C, eGFR, hs-CRP, smoking, alcohol consumption, educational level, physical activity, use of antihypertensive medication, use of hypoglycemic medication, and use of lipid-lowering medication. Participants in the MetS+CRP+ group were more likely to be older, less educated, had central obesity, higher UA, lower eGFR and higher hs-CRP than those in the MetS-CRP- group.

**Table 1:** Baseline characteristics by MetS and hs-CRP Status.

Variables	Total	MetS-CRP-	MetS-CRP+	MetS+CRP-	MetS+CRP+	P value
Participants (n)	94841	53937	10338	23521	7045	<0.001
Age,year	51.5 ± 12.5	49.6 ± 12.6	53.4 ± 13.3	53.4 ± 11.0	57.1 ± 11.1	<0.001
Male, N(%)	75976(80.0)	42529 (78.8)	8194 (78.8)	19732 (83.9)	5421 (76.9)	<0.001
SBP, mmHg	131.2 ± 21.1	125.4 ± 19.3	127.9 ± 20.9	142.0 ± 19.3	144.0 ± 20.4	<0.001
DBP, mmHg	83.6 ± 11.8	80.8 ± 10.9	81.3 ± 11.4	89.5 ± 11.2	89.3 ± 11.6	<0.001
FBG, mmol/L	5.1 (4.7–5.7)	5.0 (4.6–5.4)	4.9 (4.5–5.3)	5.8 (5.1–6.5)	5.9 (5.1–7.0)	<0.001
UA, μmol/L	289.6 ± 80.3	281.1 ± 75.1	285.8 ± 83.2	305.7 ± 83.5	306.0 ± 91.8	<0.001
WC,cm	87.0± 10.0	83.8 ± 9.4	86.8 ± 10.3	92.1 ± 7.9	94.7 ± 9.1	<0.001
TG, mmol/L	1.3 (0.9–1.9)	1.1 (0.8–1.4)	1.1 (0.8–1.5)	2.1 (1.5–3.0)	2.1 (1.5–3.0)	<0.001
TC,mmol/L	4.9 ± 1.1	4.9 ± 1.0	4.9 ± 1.0	5.1 ± 1.2	5.2 ± 1.2	<0.001
HDL-C, mmol/L	1.5 ± 0.4	1.6 ± 0.4	1.6 ± 0.4	1.5 ± 0.4	1.5 ± 0.5	<0.001
eGFR, mL/min/1.73m^2^	80.9(67.6-95.3)	82.1(69.0-96.4)	82.8(69.1-96.2)	77.7(65.1-92.7)	78.3(64.7-93.3)	<0.001
Hs-CRP,mg/L	0.8 (0.3-2.1)	0.5 (0.2-1.1)	6.1 (4.1-9.3)	0.8 (0.3-1.4)	6.1 (4.1-9.1)	<0.001
Current smoker, N (%)	32869 (34.7)	19189 (35.6)	3294 (31.9)	8332 (35.4)	2054 (29.2)	<0.001
Current drinker, N (%)	35413 (37.3)	20787 (38.5)	3365 (32.5)	9097 (38.7)	2164 (30.7)	<0.001
Physical activity,N (%)	86495 (91.2)	48983 (90.8)	9547 (92.3)	21431 (91.1)	6534 (92.7)	<0.001
High-salt Diet	10301 (10.9)	5679 (10.5)	981 (9.5)	2840 (12.1)	801 (11.4)	<0.001
Education level, N(%)						<0.001
≤junior high school	76065 (80.2)	42110 (78.1)	8264 (79.9)	19734 (83.9)	5957 (84.6)	
≥ senior high school	18776 (19.8)	11827 (21.9)	2074 (20.1)	3787 (16.1)	1088 (15.4)	
Ues of antihypertension medication, N(%)	11239 (11.9)	3554 (6.6)	986 (9.5)	4933 (21.0)	1766 (25.1)	<0.001
Use of hypoglycemic medication, N(%)	2416 (2.5)	439 (0.8)	115 (1.1)	1362 (5.8)	500 (7.1)	<0.001
Use of lipid-lowering medication, N(%)	1002 (1.1)	326 (0.6)	89 (0.9)	413 (1.8)	174 (2.5)	<0.001

SBP, systolic blood pressure; DBP, diastolic blood pressure; FBG, fasting blood glucose; UA, uric acid; WC, waist circumference; TG, triglyceride; TC, total cholesterol; LDL-C, low-density lipoprotein cholesterol; HDL-C, high-density lipoprotein cholesterol; eGFR, estimated Glomerular Filtration Rate; Hs-CRP, high-sensitivity C-reactive protein.

### Association of the combination of MetS and hs-CRP levels with the incidence of HF

**Figure 2:**
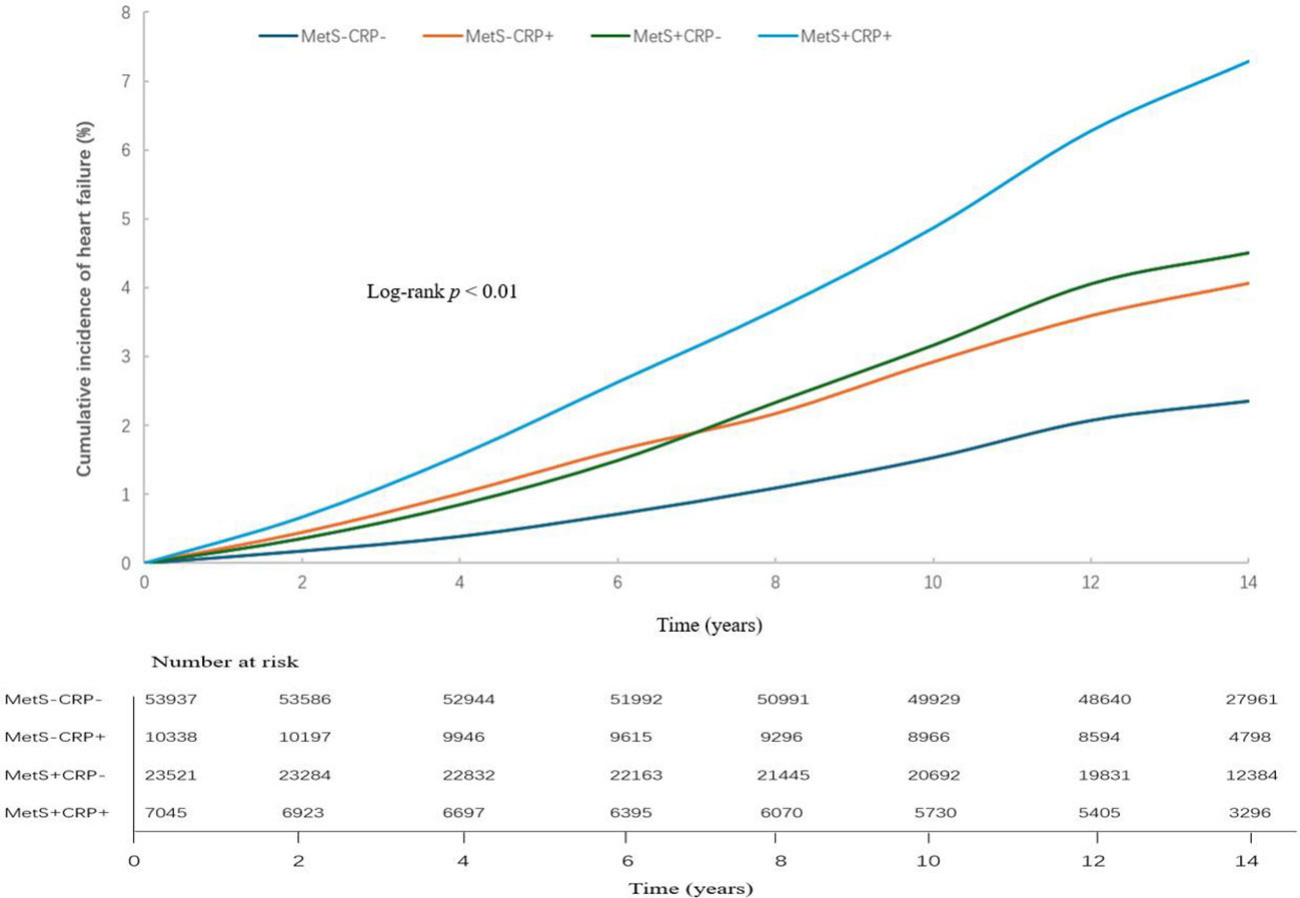
Cumulative incidence of heart failure by MetS and hs-CRP.

In studies with a mean follow-up of 13.1 ± 2.60 years, 3,058 cases of HF occurred. The cumulative incidence of HF shown in **Figure 2**. The HF incidence rate of four groups were 1.69/1000pys, 2.95/1000pys, 3.27/1000pys, 5.33/1000pys. **Table 2** showed that the risk of HF was increased by 1.29-fold (HR, 1.29; 95% CI, 1.15-1.45), 1.40-fold (HR, 1.40; 95% CI, 1.29-1.53) and 1.85-fold (HR, 1.85; 95% CI, 1.65-2.06) in the MetS-CRP+, MetS+CRP- and MetS+CRP+ groups, respectively, compared with the MetS-CRP- group. We further examined the interaction between MetS and inflammation (hs-CRP > 3 mg/L) with HF. Before adjustment for covariates, the multiplicative interaction was not statistically significant (p for interaction = 0.39), and the additive interaction metrics were statistically significant (RERI 0.49, 95% CI, 0.13- 0.85; AP 0.15, 95% CI 0.04 - 0.26; SI 1.29, 95% CI 1.07 - 1.55, P value =0.008). After adjustment for covariates, the additive interaction indicator was no longer significant (multiplicative interaction: p for interaction = 0.85, additive interaction: RERI 0.16, 95% CI, -0.08- 0.39; AP 0.08, 95% CI -0.03 - 0.20; SI 1.22, 95% CI 0.90 - 1.65, P value =0.20).

**Table 2:** Hazard ratios (HRs) for the association of MetS and hs-CRP levels with HF Risk.

Group	Case/Participants	Incidence(/1,000 person years)	HR (95%CI)		
			Model 1	Model 2	Model 3
**MetS-CRP-**	1211/53937	1.69 (1.59,1.78)	reference	reference	reference
**MetS-CRP+**	389/10338	2.95 (2.67,3.26)	1.30 (1.16,1.46)	1.30 (1.15,1.45)	1.29 (1.15,1.45)
**MetS+CRP-**	995/23521	3.27 (3.08,3.48)	1.65 (1.52,1.80)	1.59 (1.46,1.73)	1.40 (1.29,1.53)
**MetS+CRP+**	463/7045	5.33 (4.87,5.85)	2.17 (1.95,2.42)	2.09 (1.87,2.33)	1.85 (1.65,2.06)

Model 1: adjusted for age, gender;

Model 2: based on model 1,adjusted for smoking, alcohol consumption, physical activity, education, salt intake, family history of cardiovascular disease, glomerular filtration rate, uric acid;

Model 3: based on model 2, adjusted for use of antihypertensive medication, use of hypoglycemic medication, and use of lipid-lowering medication.

### Association of the MetS and components and hs-CRP with the incidence of HF

After adjustment for covariates, there was a 1.41-fold increased risk of HF in patients with MetS (HR, 1.41; 95% CI, 1.31-1.52) compared with those without MetS. In the MetS components of the HF risk study, elevated BP had the strongest association with HF (HR, 1.47; 95% CI, 1.34-1.61). The risk of HF was increased 1.29-fold (95% CI, 1.18-1.41), 1.20-fold (95% CI, 1.11-1.30), and 1.13-fold (95% CI, 1.05-1.22) for elevated WC, elevated FBG, and elevated TG, respectively. Low HDL-C levels did not show a significant effect. Those with hs-CRP levels >3 mg/L had a 1.30-fold increased risk of HF (HR, 1.30; 95% CI, 1.20-1.41) compared with those with hs-CRP levels ≤ 3 mg/L, as shown in **Table 3**.

**Table 3:** Hazard ratios (HRs) for the association of MetS and components or hs-CRP levels with HF Risk.

Group	Case/Participants	Incidence (/1,000 person years)	HR (95%CI)			
			Model 1	Model 2	Model 3	Model 4
**MetS**	1458/30566	3.73 (3.54,3.93)	1.69 (1.57,1.81)	1.62 (1.51,1.74)	1.43 (1.33,1.55)	1.41 (1.31,1.52)
**Elevated WC**	2359/60770	2.99 (2.87,3.11)	1.58 (1.46,1.72)	1.51 (1.39,1.65)	1.42 (1.30,1.55)	1.29 (1.18,1.41)
**Elevated TG**	1182/30497	2.97 (2.81,3.14)	1.39 (1.29,1.49)	1.31 (1.21,1.41)	1.24 (1.15,1.34)	1.13 (1.05,1.22)
**Low HDL-C**	271/9137	2.25 (2.00,2.53)	1.09 (0.96,1.23)	1.04 (0.92,1.18)	1.01 (0.89,1.15)	1.01 (0.89,1.14)
**Elevated BP**	2391/54195	3.45 (3.31,3.59)	1.83 (1.68,2.00)	1.83 (1.68,2.00)	1.57 (1.43,1.72)	1.47 (1.34,1.61)
**Elevated FBG**	1222/28753	3.32 (3.14,3.51)	1.42 (1.32,1.53)	1.44 (1.34,1.55)	1.29 (1.20,1.40)	1.20 (1.11,1.30)
**Hs-CRP> 3 mg/L**	852/17383	3.90 (3.65,4.17)	1.36 (1.26,1.47)	1.36 (1.25,1.47)	1.34 (1.23,1.45)	1.30 (1.20,1.41)

Model 1: adjusted for age, gender;

Model 2: based on model 1, adjusted for smoking, alcohol consumption, physical activity, education, salt intake, family history of cardiovascular disease, glomerular filtration rate, uric acid;

Model 3: based on model 2, adjusted for use of antihypertensive medication, use of hypoglycemic medication, and use of lipid-lowering medication;

Model 4: MetS: based on model 3, adjusted for hs-CRP; MetS components: based on model 3, adjusted for hs-CRP and other components; Hs-CRP: based on model 3, adjusted for MetS.

### Age and sex stratified analysis of the association of the combination of MetS and hs-CRP levels with the risk of HF

There was an interaction between groups and age (p for interaction < 0.01). Compared with MetS-CRP-, the risk of HF in the MetS+CRP+ group was increased 2.17-fold (HR, 2.17; 95% CI, 1.83-2.57) in participants with < 60 years and 1.53-fold (HR, 1.53; 95% CI, 1.32-1.78) in participants with ≥ 60 years. Lifestyle and medication information grouped by age were shown in Supplementary Material **Supplementary Table S1**. There was no interaction between groups and sex (p for interaction >0.05). Compared with MetS-CRP-, the risk of HF in the MetS+CRP+ group was 1.81-fold (HR, 1.81; 95% CI, 1.60-2.04) in males and 1.93-fold (HR, 1.93; 95% CI, 1.47-2.53) in females, respectively. The results were shown in **Table 4**.

**Table 4:** Hazard ratios (HRs) for the association of MetS and hs-CRP levels with HF risk by age and gender.

Group	Case/Participants	Incidence (/1, 000 person years)		HR(95%CI)
Age<60years				
**MetS-CRP-**	584/44103	0.97 (0.89,1.05)	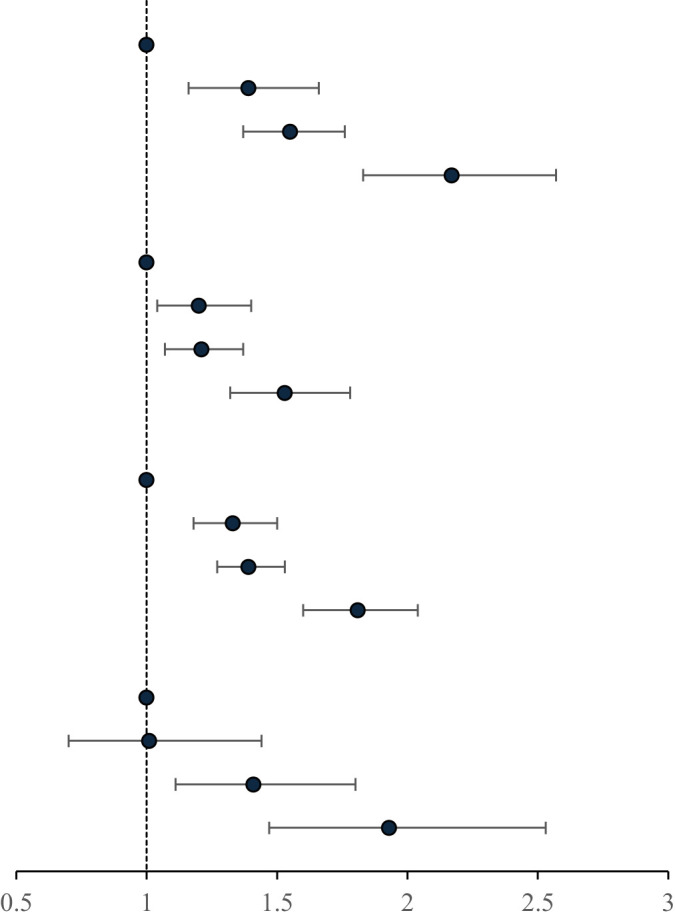	reference
**MetS-CRP+**	148/7221	1.53 (1.30,1.80)		1.39 (1.16,1.66)
**MetS+CRP-**	522/17781	2.19 (2.01,2.39)		1.55 (1.37,1.76)
**MetS+CRP+**	199/4454	3.41 (2.97,3.92)		2.17 (1.83,2.57)
Age>=60years				
**MetS-CRP-**	627/9834	5.36 (4.96,5.80)		reference
**MetS-CRP+**	241/3117	6.88 (5.06,7.80)		1.20 (1.04,1.40)
**MetS+CRP-**	473/5740	7.18 (6.57,7.86)		1.21 (1.07,1.37)
**MetS+CRP+**	264/2591	9.31 (8.26,10.51)		1.53 (1.32,1.78)
Male				
**MetS-CRP-**	1060/42529	1.89 (1.78,2.00)		reference
**MetS-CRP+**	352/8194	3.43 (3.09,3.80)		1.33 (1.18,1.50)
**MetS+CRP-**	840/19732	3.31(3.09,3.54)		1.39 (1.27,1.53)
**MetS+CRP+**	364/5421	5.55(5.01,6.15)		1.81 (1.60,2.04)
Female				
**MetS-CRP-**	151/11408	0.96 (0.82,1.13)		reference
**MetS-CRP+**	37/2144	1.27 (0.92,1.76)		1.01 (0.70,1.44)
**MetS+CRP-**	155/3789	3.08 (2.63,3.61)		1.41 (1.11,1.80)
**MetS+CRP+**	99/1624	4.68 (3.84,5.70)		1.93 (1.47,2.53)

The model adjusted for age, gender, smoking, alcohol consumption, physical activity, education, salt intake, family history of cardiovascular disease, glomerular filtration rate, uric acid, use of antihypertensive medication, use of hypoglycemic medication, and use of lipid-lowering medication.

### Antihypertensive drugs stratified analysis of the association of the combination of MetS and hs-CRP levels with the risk of HF

There was an interaction between groups and ues of antihypertension medication (p for interaction <0.01). Compared with MetS-CRP-, the risk of HF in the MetS+CRP+ group was increased 1.38-fold (HR, 1.38; 95% CI, 1.12-1.70) in participants with antihypertension medication use and 2.00-fold (HR, 2.00; 95% CI, 1.75-2.27) in participants without antihypertension medication use. We continued to stratify according to the intensity of treatment, divided into monotherapy and combination therapy (at least two types of antihypertensive drugs). After stratification, the HR (HR, 1.27; 95% CI, 1.04-1.56) of the combined treatment of MetS + CRP + group was significantly lower than that of the single drug treatment of MetS + CRP + group (HR, 1.76; 95% CI, 1.26-2.48). The results were shown in **Table 5**.

**Table 5:** Hazard ratios (HRs) for the association of MetS and hs-CRP levels with HF risk by antihypertensive medication.

Group	Case/Participants	Incidence (/1, 000 person years)		HR (95%CI)
With Medication				
**MetS-CRP-**	222/3554	5.05 (4.43,5.76)	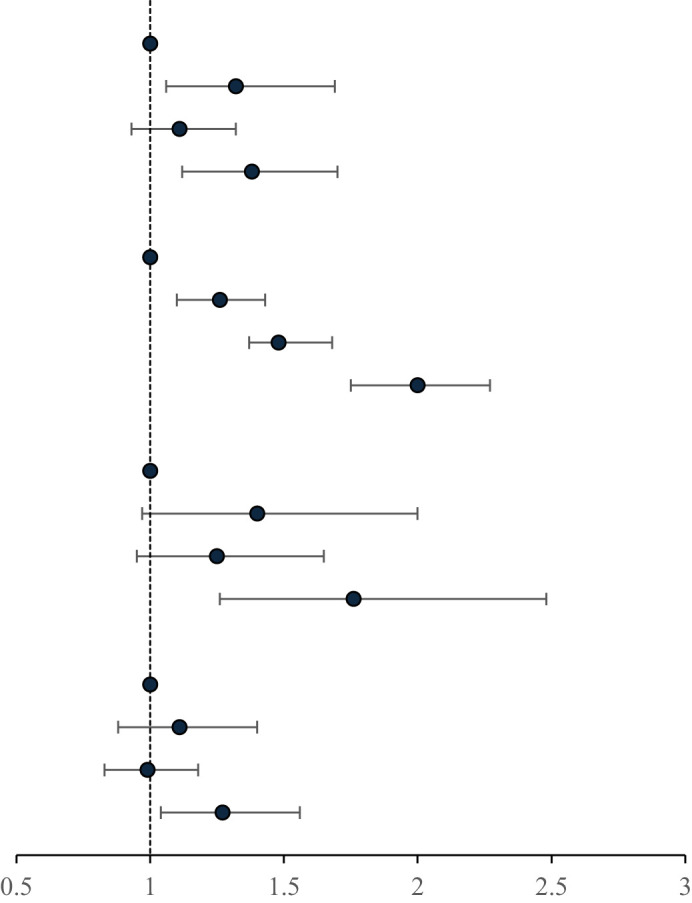	reference
**MetS-CRP+**	89/986	7.76 (6.30,9.55)		1.32 (1.03,1.69)
**MetS+CRP-**	345/4933	5.65 (5.08,6.28)		1.11 (0.93,1.32)
**MetS+CRP+**	159/1766	7.69 (6.58,8.98)		1.38 (1.12,1.70)
Without Medication				
**MetS-CRP-**	1989/50383	1.47 (1.38,1.56)		reference
**MetS-CRP+**	300/9352	2.49 (2.23,2.79)		1.26 (1.10,1.43)
**MetS+CRP-**	650/18588	2.68 (2.48,2.89)		1.48 (1.37,1.68)
**MetS+CRP+**	304/5279	4.60 (4.11,5.15)		2.00 (1.75,2.27)
Monotherapy				
**MetS-CRP-**	110/2395	3.74 (3.10,4.51)		reference
**MetS-CRP+**	40/601	5.97 (4.38,8.14)		1.40 (0.97,2.00)
**MetS+CRP-**	100/1625	5.06 (4.16,6.16)		1.25 (0.95,1.65)
**MetS+CRP+**	51/573	7.72 (5.87,10.16)		1.76 (1.26,2.48)
Combination Therapy				
**MetS-CRP-**	255/2067	11.22 (9.93,12.69)		reference
**MetS-CRP+**	96/684	13.85 (11.34,16.92)		1.11 (0.88,1.40)
**MetS+CRP-**	284/2297	11.19 (9.96,12.57)		0.99 (0.83,1.18)
**MetS+CRP+**	153/957	15.68 (13.39,18.38)		1.27 (1.04,1.56)

The model adjusted for age, gender, smoking, alcohol consumption, physical activity, education, salt intake, family history of cardiovascular disease, glomerular filtration rate, uric acid, use of hypoglycemic medication, and use of lipid-lowering medication.

### Sensitivity analysis

We separately excluded participants who had MI (N=403), AF (N=204) before the onset of HF, and the results remained robust after adjustment for covariates. We redefined the MetS population using the IDF definition of MetS and the results remained similar. CRP was grouped at 2 mg/L and the results were consistent with the main analysis. We separately excluded those with hs-CRP >10 mg/L (N=3,682), those with pre-diabetes (N=19,903), those with hypertension (N=42,287), those with diabetes (N=9,059), those with dyslipidemia (N=33,444), those on medication (N=12,878), and those who progressed to MetS (N=33,261), and the results were consistent with the primary outcome. Due to baseline age differences among the four groups, we used matching, and the results were consistent with the main results. The baseline characteristics of the four groups after matching were shown in Supplementary Material: **Supplementary Table S2**. The results were shown in **Table 6**.

**Table 6:** Sensitivity analysis.

Group	Case/Participants	Incidence (/1, 000 person years)		HR(95%CI)
Sensitivity 1				
**MetS-CRP-**	1083/53809	1.51 (1.42,1.60)	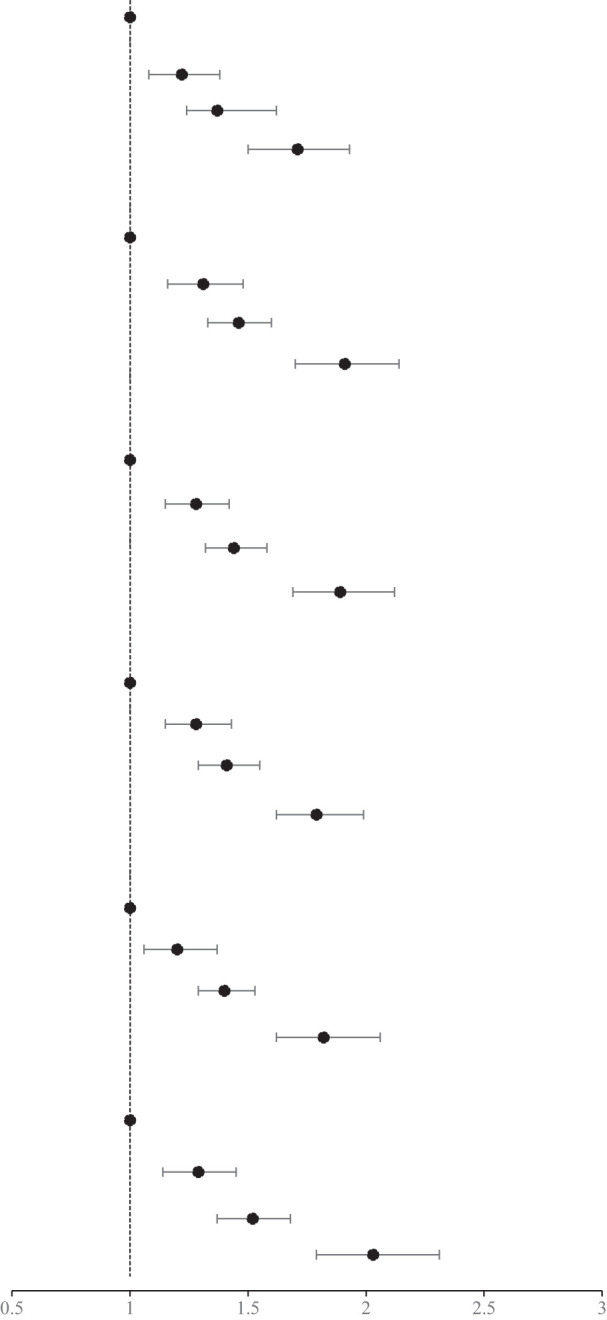	reference
**MetS-CRP+**	328/10277	2.50 (2.24,2.78)		1.22 (1.08,1.38)
**MetS+CRP-**	863/22388	2.85 (2.66,3.04)		1.37 (1.25,1.50)
**MetS+CRP+**	382/6964	4.43 (4.01,4.90)		1.71 (1.52,1.93)
Sensitivity 2				
**MetS-CRP-**	1112/53838	1.55 (1.46,1.64)		reference
**MetS-CRP+**	361/10310	2.74 (2.47,3.04)		1.31 (1.16,1.48)
**MetS+CRP-**	946/23472	3.12 (2.92,3.32)		1.46 (1.33,1.60)
**MetS+CRP+**	435/7017	5.03 (4.58,5.53)		1.91 (1.70,2.14)
Sensitivity 3				
**MetS-CRP-**	1417/60697	1.76 (1.67,1.85)		reference
**MetS-CRP+**	441/11840	3.02 (2.75,3.32)		1.28 (1.15,1.42)
**MetS+CRP-**	789/16761	3.65 (3.41,3.92)		1.44 (1.32,1.58)
**MetS+CRP+**	411/5903	5.66 (5.14,6.24)		1.89 (1.69,2.12)
Sensitivity 4				
**MetS-CRP-**	1083/49756	1.63 (1.54,1.73)		reference
**MetS-CRP+**	517/14519	2.78 (2.55,3.03)		1.28 (1.15,1.43)
**MetS+CRP-**	842/20637	3.14 (2.94,3.36)		1.41 (1.29,1.55)
**MetS+CRP+**	616/9929	5.01 (4.63,5.42)		1.79 (1.62,1.99)
Sensitivity 5				
**MetS-CRP-**	1211/53937	1.69 (1.59,1.78)		reference
**MetS-CRP+**	283/8088	2.73 (2.43,3.06)		1.20 (1.06,1.37)
**MetS+CRP-**	995/23521	3.27 (3.08,3.48)		1.40 (1.29,1.53)
**MetS+CRP+**	367/5613	5.28 (4.77,5.85)		1.82 (1.62,2.06)
Sensitivity 6				
**MetS-CRP-**	1100/47315	1.75 (1.65,1.85)		reference
**MetS-CRP+**	357/9262	3.03 (2.73,3.36)		1.29 (1.14,1.45)
**MetS+CRP-**	679/13951	3.79 (3.51,4.09)		1.52 (1.37,1.68)
**MetS+CRP+**	326/4410	6.06 (5.44,6.76)		2.03(1.79,2.31)
	
Sensitivity 7				
**MetS-CRP-**	575/37586	1.13 (1.04,1.23)	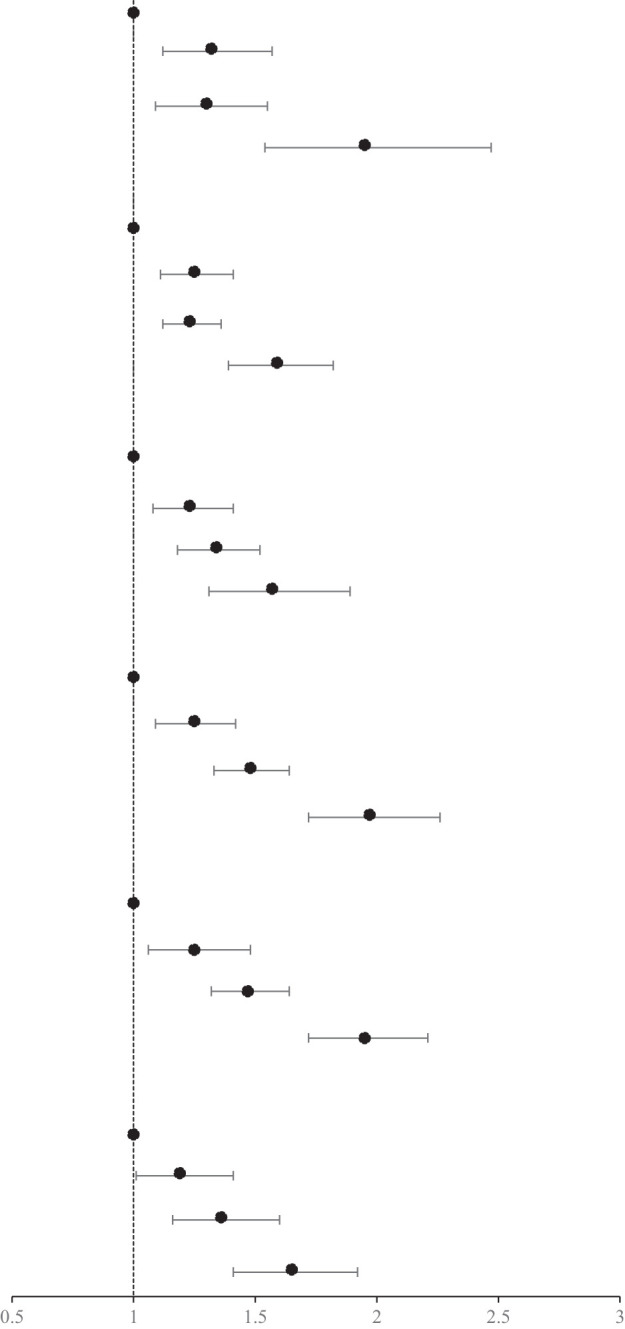	reference
**MetS-CRP+**	180/6561	2.10 (1.82,2.43)		1.32 (1.12,1.57)
**MetS+CRP-**	169/6707	1.89 (1.63,2.20)		1.30 (1.09,1.55)
**MetS+CRP+**	80/1700	3.67 (2.94,4.56)		1.95 (1.54,2.47)
Sensitivity 8				
**MetS-CRP-**	1129/52229	1.62 (1.53,1.72)		reference
**MetS-CRP+**	361/9927	2.84 (2.56,3.15)		1.25 (1.11,1.41)
**MetS+CRP-**	634/18525	2.61 (2.41,2.82)		1.23 (1.12,1.36)
**MetS+CRP+**	277/5101	4.34 (3.86,4.88)		1.59 (1.39,1.82)
Sensitivity 9				
**MetS-CRP-**	900/42000	1.61 (1.51,1.72)		reference
**MetS-CRP+**	275/7966	2.70 (2.40,3.04)		1.23 (1.08,1.41)
**MetS+CRP-**	342/8806	3.02 (2.72,3.36)		1.34 (1.18,1.52)
**MetS+CRP+**	142/2625	4.42 (3.75,5.21)		1.57 (1.31,1.89)
Sensitivity 10				
**MetS-CRP-**	961/49861	1.44 (1.35,1.53)		reference
**MetS-CRP+**	290/9208	2.45 (2.18,2.74)		1.25 (1.09,1.42)
**MetS+CRP-**	588/17863	2.51 (2.32,2.72)		1.48 (1.33,1.64)
**MetS+CRP+**	269/5031	4.26 (3.78,4.80)		1.97 (1.72,2.26)
Sensitivity 11				
**MetS-CRP-**	588/26232	reference	
**MetS-CRP+**	181/4782	3.14 (2.71,3.63)		1.25 (1.06,1.48)
**MetS+CRP-**	995/23521	3.27 (3.08,3.48)		1.47 (1.32,1.64)
**MetS+CRP+**	463/7045	5.34 (4.87,5.85)		1.95 (1.72,2.21)
Sensitivity 12				
**MetS-CRP-**	261/7045	2.87 (2.54,3.24)		reference
**MetS-CRP+**	304/7045	3.42 (3.06,3.83)		1.19 (1.01,1.41)
**MetS+CRP-**	396/7044	4.48 (4.06,4.94)		1.36 (1.16,1.60)
**MetS+CRP+**	463/7045	5.34 (4.87,5.85)		1.65 (1.41,1.92)
	

The model adjusted for age, gender, smoking, alcohol consumption, physical activity, education, salt intake, family history of cardiovascular disease, glomerular filtration rate, uric acid, use of antihypertensive medication, use of hypoglycemic medication, and use of lipid-lowering medication.

Sensitivity 1: excluded participants who had myocardial infarction before the onset of heart failure (N=403). Sensitivity 2: excluded participants who had atrial fibrillation before the onset of heart failure (N=204). Sensitivity 3: Using the IDF definition of metabolic syndrome. Sensitivity 4: CRP grouped by 2 mg/L. Sensitivity 5: excluded participants with CRP >10 mg/L (N=3,682). Sensitivity 6: excluded participants with pre-diabetes (N=19,903). Sensitivity 7: excluded participants with hypertension (N=42,287). Sensitivity 8: excluded participants with diabetes (N=9,059). Sensitivity 9: excluded participants with dyslipidemia (N=33,444). Sensitivity 10: excluded participants on medication (N=12,878). Sensitivity 11: excluded participants who progressed to MetS (N=33,261). Sensitivity 12: using matching method.

## Discussion

The results showed that the combination of MetS and high hs-CRP increased the risk of HF. Our stratified analysis found that in young people, people with metabolic disorders combined with inflammation had a higher relative risk of HF compared to people with metabolically healthy non-inflammation. This study highlighted the potential importance of MetS and inflammation as part of a strategy to prevent HF, especially when both are present. Especially in young people, early prevention and intervention may have more significant long-term benefits.

Several mechanisms may explain the association between MetS and inflammation with HF. Insulin resistance is the main pathogenesis of MetS (25). Chronic inflammation released pro-inflammatory cytokines such as TNF-α and IL-6, which affect insulin signalling pathways and lead to insulin resistance (26). The coexistence of both exacerbated insulin resistance and impaired two signalling pathways, the phosphatidylinositol 3-kinase (PI3K)/protein kinase B (Akt) and mitogen-activated protein kinase (MAPK) pathways, leading to cardiomyocyte apoptosis and fibrosis (27). Systemic inflammation in patients with MetS also led to activation of the vegetative angiotensin system and the sympathetic nervous system, resulting in volume expansion, increased peripheral resistance, and compensatory myocardial enlargement (7). In addition, the onset of MetS and the binding of CRP to CD32 and CD64 receptors on endothelial cells triggered pro-inflammatory pathways (28). These pathways might increase susceptibility to HF.

We found that the association of MetS with HF was stronger than the association of CRP with HF and provided several possible explanations. First, elevated BP in the MetS component was most strongly associated with HF. According to the 2019 GBD data, the main causes of HF worldwide are ischemic heart disease and hypertensive heart disease. Among them, the proportion of HF caused by hypertensive heart disease was 32.84% (29). Hypertension was a key component of MetS. In China, the awareness, treatment and compliance rates of hypertensive patients were still low compared with those in developed countries (30). Therefore, effective management of hypertension in MetS has become an important direction for improving HF prevention strategies. Second, after we excluded the hypertensive and diabetes populations from the sensitivity analyses, we found that the association between CRP and HF was slightly stronger than that between MetS and HF. This indicated that MetS with combined hypertension and hyperglycemia played a key role in the pathogenesis of HF. The additive effect of MetS combined with hypertension and hyperglycemia on the risk of developing HF was unclear and may be related to activation of insulin resistance and chronic inflammation. Last, the association of CRP with HF with preserved ejection fraction (HFpEF) and with reduced ejection fraction (HFrEF) was controversial. Several studies showed a stronger association between CRP and HFpEF (28, 31). In another study, CRP was independently associated with HFrEF but not with HFpEF (32). However, we were unable to typify due to missing data on ejection fraction. The association between CRP and HF might be underestimated by the different proportions of HF in different types.

After stratified for age, we found that the MetS+CRP+ group had a higher relative risk of HF in those <60 years of age. According to previous studies, the relative risk of cardiovascular disease differed between different age groups for the onset of MetS, hypertension, and diabetes, and this association was particularly strong in the younger population (33–35). Although the incidence and absolute risk of HF were higher in older populations, modifiable clinical risk factors had higher relative and population-attributable risks in younger populations (36). People with early onset of metabolic disease more frequently had concomitant obesity and poor risk factor control (37). Younger people were less likely to take interventions for hypertension, diabetes, etc., and were more likely than older people to have unhealthy lifestyles, which may have increased their relative risk of developing HF. We compared lifestyles and drug use in different age groups and found that the proportion of unhealthy lifestyles such as smoking and drinking was higher in people under 60 years old, and although most of them maintained exercise habits, they may still increased the relative risk of HF due to poor health management. In contrast, people over 60 years old were more inclined to maintain healthy lifestyle habits. Although they had a high prevalence of chronic diseases, they were more active in receiving drug treatment.

Our results found that antihypertensive therapy alleviated the association between participants with metabolic disorders combined with inflammation and the risk of HF, and that combination therapy was more significant than monotherapy. At present, no drugs have been approved for the treatment of MetS itself (38). Our results suggested the importance of targeting antihypertensive treatment to participants with metabolic disorders associated with inflammation. Elevated CRP in patients with MetS might predicted worse subclinical impairment (39, 40), and the included of CRP in the risk assessment of patients with MetS might identified high-risk subgroups who might benefit from intensive antihypertensive therapy.

We did several sensitivity analyses. The MI and AF cause HF (14, 41, 42). We excluded participants with MI and AF that occurred before HF, respectively, and the associations were not significantly changed. It has been suggested that chronic low-grade inflammation be defined as CRP > 2 mg/L (28). Therefore, we repeated the primary analysis and the results were consistent. The definition of MetS included people with pre-diabetes. Studies reported that pre-diabetes increased the risk of HF (43). We excluded this group, and the results remained robust. We observed that the change of HF risk was small after excluding pre-diabetes, and was significantly lower than that of excluding diabetes. Some studies have reported that the more elevated FBG levels, the more likely they are to adversely affect the heart by inducing inflammation, modulating nitric oxide metabolism and increasing oxidative stress (44). These differences may lead to less myocardial damage in prediabetes than in diabetes. In a prospective cohort study of 18,084 patients with cardiovascular disease, a 1 mmol/L increased in FBG was associated with a 1.23-fold increased risk of hospitalisation for HF (45). Individuals without MetS at baseline might progress to MetS during follow-up. Studies showed that the occurrence of MetS, even if only once, might have had long-term adverse effects on cardiovascular health, and this effect was difficult to completely reverse regardless of whether the individual eventually recovered (24). Therefore, we excluded people who had MetS during follow-up, and the results did not change.

It provided a unique perspective on the association of MetS and inflammation with the risk of HF onset. The age range of participants in this study was 18-90 years, which was well represented. Despite these strengths, it was important to acknowledge certain limitations of the study. First, the use of a single biomarker for baseline measurements was a potential limitation. However, the large sample size mitigated this limitation to some extent. In addition, we did not measure other inflammatory markers such as IL-6. We lacked indicators that reflect early organ damage. For example, the Mechanical Energy Efficiency Index (MEEI) was a valuable predictor of HF (46), and elevated CRP, MetS exacerbated myocardial MEEI (39). We lacked data on ejection fraction, and the association of MetS and inflammation with HF needed to be further discussed in the different subtypes of HF.

## Conclusion

In the Chinese population, the combination of MetS and high hs-CRP levels increased the risk of HF, especially in young people. Early intervention for metabolic abnormalities combined with inflammation might prevent the development of HF.

## Data Availability

The raw data supporting the conclusions of this article will be made available by the authors, without undue reservation.
