# Supplementary material for: Metabolic syndrome, high-sensitivity C-reactive protein and the risk of heart failure: the Kailuan cohort study
Source: Front Endocrinol (Lausanne). 2025 Apr 22;16:1544823. doi: 10.3389/fendo.2025.1544823 (PMC12053502; doi:10.3389/fendo.2025.1544823)
Supplement: Supplementary file 1 [file DataSheet1.pdf]

Table S1 Lifestyle and Medication Information Grouped by Age

variables	Total	< 60 years	≥ 60 years	P value
Participants (n)	94841	73559	21282	
Current smoker, N (%)	32869(34.7)	27073(36.8)	5769(27.2)	<0.001
Current drinker, N (%)	35413(37.3)	29221(39.7)	6192(29.1)	<0.001
Physical activity, N (%)	86495(91.2)	66263(90.1)	20232(95.1)	<0.001
High-salt Diet, N (%)	10301(10.9)	7968(10.8)	2333(11.0)	0.59
Hypertension, N (%)	42287(44.6)	28878(39.3)	13409(63.0)	<0.001
Ues of antihypertension medication, N(%)	11239(11.9)	6555(8.9)	4684(22.0)	<0.001
Diabetes, N (%)	9059(9.6)	5961(8.1)	3098(14.6)	<0.001
Use of hypoglycemic medication, N(%)	2416(2.5)	1323(1.8)	1093(5.1)	<0.001
Dyslipidemia, N (%)	33444(35.3)	25683(34.9)	7761(36.5)	<0.001
Use of lipid-lowering medication, N(%)	1002(1.1)	577(0.8)	425(2.0)	<0.001

Table S2 Baseline Characteristics by MetS and hs-CRP Status in Matched Population

variables	Total	MetS-CRP-	MetS-CRP+	MetS+CRP-	MetS+CRP+	P value
Participants (n)	28179	7045	7045	7044	7045	
Age,year	57.1 ± 11.2	57.1 ± 11.2	57.1± 11.2	57.1 ± 11.2	57.1 ± 11.2	0.99
Male, N(%)	22819(81.0)	5808 (82.4)	5696 (80.9)	5894 (83.7)	5421 (76.9)	<0.001
SBP, mmHg	136.7 ± 21.7	129.7 ± 20.8	129.8 ± 21.3	143.5 ± 19.5	144.0 ± 20.4	<0.001
DBP, mmHg	85.6 ± 11.9	81.9 ± 11.0	81.9 ± 11.5	89.1± 11.2	89.3 ± 11.6	<0.001
FBG, mmol/L	5.3 (4.7–6.0)	5.0 (4.6–5.4)	4.9 (4.5–5.4)	5.8 (5.1–6.6）	5.9 (5.1–7.0)	<0.001
UA, µmol/L	296.4 ± 85.2	286.2 ± 75.8	286.1 ± 84.6	307.4 ± 85.3	306.0 ± 91.8	<0.001
WC,cm	89.8 ± 10.0	84.8 ± 9.4	87.1 ± 10.1	92.3 ± 8.0	94.7 ± 9.1	<0.001
TG, mmol/L	1.5 (1.0–2.2)	1.1 (0.8–1.4)	1.1 (0.8–1.5)	2.0 (1.4–2.8)	2.1 (1.5–3.0)	<0.001
TC,mmol/L	5.0 ± 1.1	5.0 ± 1.0	4.9 ± 1.0	5.1 ± 1.2	5.2 ± 1.2	<0.001
HDL-C, mmol/L	1.6 ± 0.4	1.6 ± 0.4	1.6 ± 0.4	1.5 ± 0.4	1.5 ± 0.5	<0.001
eGFR, mL/min/1.73m^2	78.1(65.3-92.3)	77.9(65.6-91.2)	81.2(68.1-94.4)	75.2(63.0-89.9)	78.3(64.7-93.3)	<0.001
Hs-CRP,mg/L	3.0 (0.7-6.2)	0.6 (0.2-1.1)	6.2 (4.1-9.3)	0.8 (0.4-1.5)	6.1 (4.1-9.1)	<0.001
Current smoker, N (%)	8937 (31.7)	2440 (34.6)	2211 (31.4)	2232 (31.7)	2054 (29.2)	<0.001
Current drinker, N (%)	9342 (32.2)	2478 (35.2)	2179 (30.9)	2521 (35.8)	2164 (30.7)	<0.001
Physical activity, N (%)	26088 (92.6)	6509 (92.4)	6535 (92.8)	6510 (92.4)	6534 (92.7)	0.74
High-salt Diet,N (%)	3053 (10.8)	753 (10.7)	667 (9.5)	832 (11.8)	801 (11.4)	<0.001
Education level, N(%)						<0.001
≤junior high school	23740 (84.2)	5902 (83.8)	5929 (84.2)	5952 (84.5)	5957 (84.6)	
≥ senior high school	4439 (15.8)	1143 (16.2)	1116 (15.8)	1092 (15.5)	1088 (15.4)	
Ues of antihypertension medication, N(%)	4866 (17.3)	685 (9.7)	787 (11.2)	1628 (23.1)	1766 (25.1)	<0.001
Use of hypoglycemic medication, N(%)	1155 (4.1)	79 (1.1)	89 (1.3)	487 (6.9)	500 (7.1)	<0.001
Use of lipid-lowering medication, N(%)	460 (1.6)	71 (1.0)	68 (1.0)	147 (2.1)	174 (2.5)	<0.001

Abbreviations: SBP, systolic blood pressure; DBP, diastolic blood pressure; FBG, fasting blood glucose; UA , uric acid; WC, waist circumference; TG, triglyceride; TC, total cholesterol; LDL-C, low-density lipoprotein cholesterol; HDL-C, high-density lipoprotein cholesterol; eGFR, estimated Glomerular Filtration Rate; Hs-CRP, high-sensitivity C-reactive protein.
